# Direct observations of anomalous resistivity and diffusion in collisionless plasma

**DOI:** 10.1038/s41467-022-30561-8

**Published:** 2022-05-26

**Authors:** D. B. Graham, Yu. V. Khotyaintsev, M. André, A. Vaivads, A. Divin, J. F. Drake, C. Norgren, O. Le Contel, P.-A. Lindqvist, A. C. Rager, D. J. Gershman, C. T. Russell, J. L. Burch, K.-J. Hwang, K. Dokgo

**Affiliations:** 1 Swedish Institute of Space Physics, Uppsala, Sweden; 2 Space and Plasma Physics, School of Electrical Engineering and Computer Science, KTH Royal Institute of Technology, Stockholm, Sweden; 3 Faculty of Physics, Earth Physics Department, Saint Petersburg State University, Saint Petersburg, Russia; 4 IREAP, University of Maryland, College Park, MD USA; 5 Department of Physics and Technology, University of Bergen, Bergen, Norway; 6 Laboratoire de Physique des Plasmas, UMR7648, CNRS/Ecole Polytechnique/Sorbonne Université/Univ. Paris Sud/Observatoire de Paris, Paris, France; 7 NASA Goddard Space Flight Center, Greenbelt, MD USA; 8 Department of Physics, Catholic University of America, Washington, DC USA; 9 Department of Earth and Space Sciences, University of California, Los Angeles, CA USA; 10 Southwest Research Institute, San Antonio, TX USA

**Keywords:** Magnetospheric physics, Astrophysical plasmas

## Abstract

Coulomb collisions provide plasma resistivity and diffusion but in many low-density astrophysical plasmas such collisions between particles are extremely rare. Scattering of particles by electromagnetic waves can lower the plasma conductivity. Such anomalous resistivity due to wave-particle interactions could be crucial to many processes, including magnetic reconnection. It has been suggested that waves provide both diffusion and resistivity, which can support the reconnection electric field, but this requires direct observation to confirm. Here, we directly quantify anomalous resistivity, viscosity, and cross-field electron diffusion associated with lower hybrid waves using measurements from the four Magnetospheric Multiscale (MMS) spacecraft. We show that anomalous resistivity is approximately balanced by anomalous viscosity, and thus the waves do not contribute to the reconnection electric field. However, the waves do produce an anomalous electron drift and diffusion across the current layer associated with magnetic reconnection. This leads to relaxation of density gradients at timescales of order the ion cyclotron period, and hence modifies the reconnection process.

## Abstract (web summary)

It is suggested that waves can provide both diffusion and resistivity that can potentially support the reconnection electric field in low-density astrophysical plasmas. Here, the authors show, using direct spacecraft measurements, that the waves contribute to anomalous diffusion but do not contribute to the reconnection electric field.

## Introduction

Most of the visible universe is composed of plasma, consisting of ions and electrons. The behavior of plasma is governed by electromagnetic forces. In low-density solar and astrophysical plasmas Coulomb collisions are typically extremely rare, meaning that collisions between particles do not play a role in the behavior of the plasma and cannot provide plasma resistivity and diffusion. However, the scattering of particles by electromagnetic waves can introduce effective collisions, lowering the plasma conductivity^1,2^. Such anomalous resistivity due to wave-particle interactions is thought to be crucial to a wide variety of collisionless plasma processes^3–5^. One process where anomalous effects are thought to be important is magnetic reconnection, which is a fundamental plasma process providing explosive energy releases by reconfiguring magnetic field topology^6,7^. In particular, it has been suggested based on theoretical and numerical results that waves can provide both diffusion and resistivity, which can potentially support the reconnection electric field^8,9^, the out-of-plane electric field responsible for sustaining reconnection.

One wave that has received significant attention as a source of anomalous effects is the lower hybrid wave^10–12^. Lower hybrid waves are found at frequencies between the ion and electron cyclotron frequencies and are driven by plasma gradients and the associated cross-field currents^11,13^. Previous attempts to calculate anomalous terms concluded that the anomalous resistivity was small^14,15^, while cross-field particle diffusion associated could be significant^16,17^. However, these estimates relied on density fluctuations inferred from the spacecraft potential, electron velocities inferred from the electric and magnetic fields assuming electrons remain frozen in, and often single spacecraft measurements. An external electric field can modify the spacecraft potential, making density fluctuations associated with waves inferred from the spacecraft potential unreliable^18,19^. Similarly, it is unclear how well the frozen-in approximation works without direct measurements. Recent observations have shown that electrons remain close to frozen in, although pressure fluctuations associated with the waves can cause some deviation from the ideal frozen in condition^20^. Thus, calculations of anomalous resistivity, viscosity, and cross-field diffusion based on direct particle measurements are needed to determine the role of lower hybrid waves.

In this work, we directly measure and quantify anomalous resistivity, viscosity, and cross-field electron diffusion associated with lower hybrid waves using the high-resolution fields and particle measurements from the four MMS spacecraft^21^. We show that anomalous resistivity (drag) is balanced by viscosity (momentum transport), and thus the waves do not contribute to the reconnection electric field. However, the waves do produce an anomalous electron drift and diffusion across the current layer associated with magnetic reconnection. This can lead to the relaxation of density gradients at timescales of order the ion-cyclotron period, which counteracts steepening of density gradients caused by magnetic reconnection and hence modifies the process.

## Results

### Magnetic reconnection and case study

**Fig. 1:**
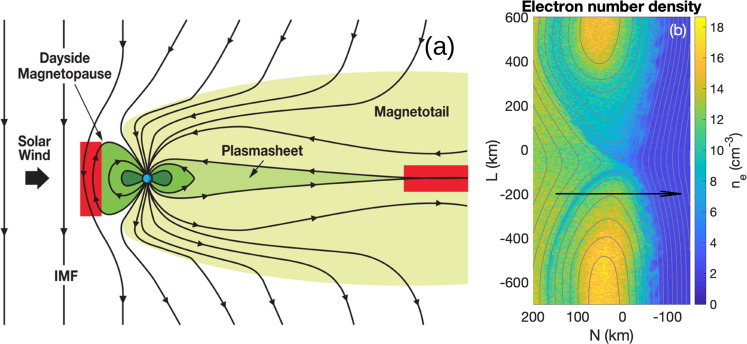
Magnetic reconnection at the magnetopause. Sketch of the magnetosphere (from https://mms.gsfc.nasa.gov/science.html) and a numerical simulation, showing an overview of a region with lower hybrid waves, anomalous plasma effects, and magnetic reconnection. **a** Sketch of the magnetosphere around Earth (blue circle) and the regions where magnetic reconnection is expected to occur (indicated by red-shaded regions). The black lines show the magnetic field lines associated with the interplanetary magnetic field (IMF) and Earth's magnetosphere (indicated by the green-shaded regions). The dark green region corresponds to the Van Allen radiation belt region. Magnetic reconnection occurs at the magnetopause, the boundary between higher-density solar wind/magnetosheath and lower-density magnetospheric plasma. **b** Three-dimensional simulation of magnetic reconnection at the magnetopause (see Methods, subsection Simulation description for details on the simulation parameters). Reconnection at the magnetopause is asymmetric, meaning the upstream conditions on the left and right differ significantly. The gray lines indicate the magnetic field lines and the color shading indicates electron density *n*_*e*_. Lower hybrid waves at the density gradient drive fluctuations in *n*_*e*_, which can cause significant electron diffusion and broadening of the layer, consistent with MMS observations. The black arrow indicates the approximate MMS trajectory through the reconnection event in Fig. 2 based on MMS observations (see ref. ^28^). The parameters used in the simulation are based on this reconnection event.

**Fig. 2:**
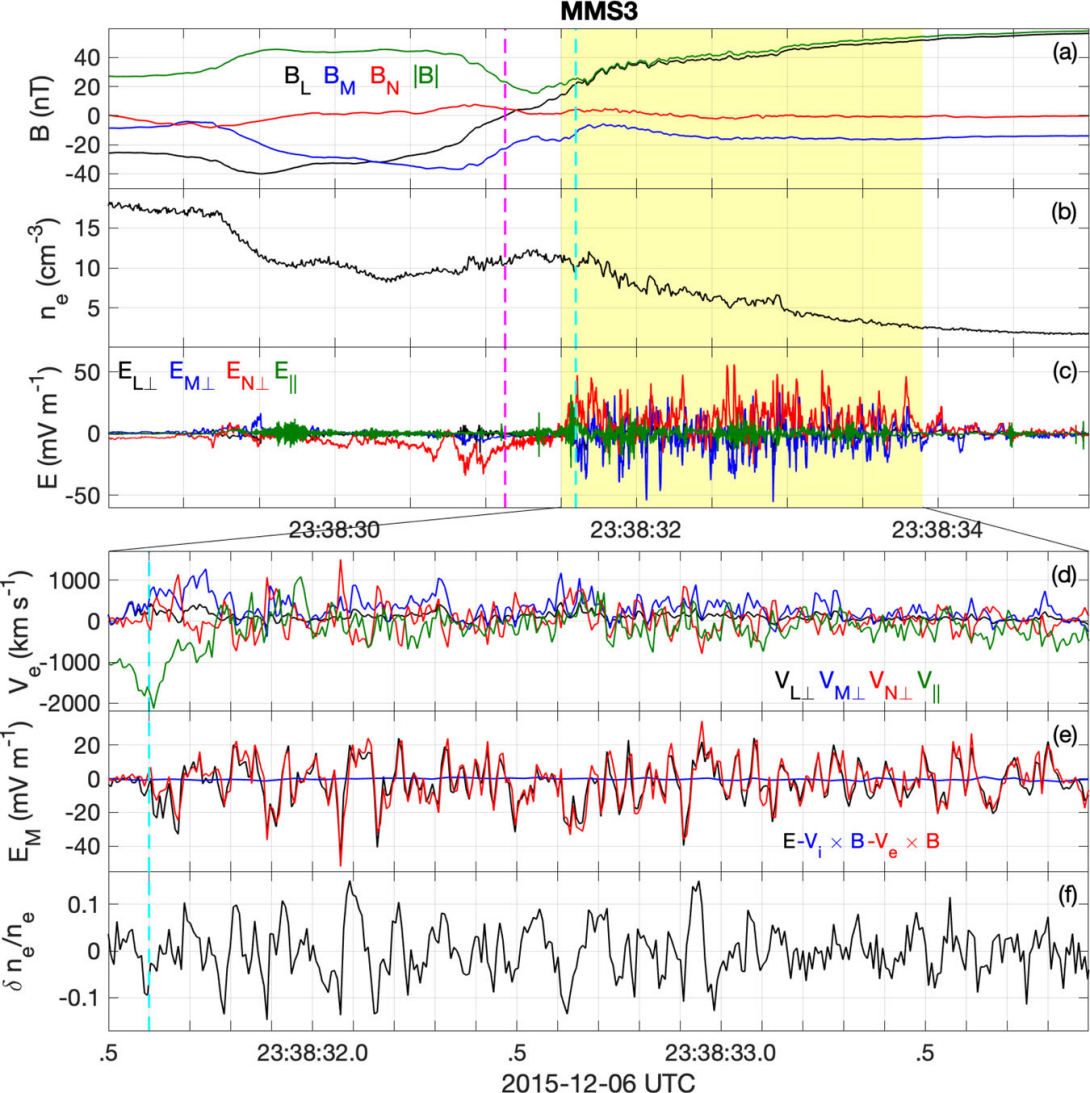
Magnetopause crossing. Observations of the magnetopause current sheet by MMS3, including lower hybrid waves and associated electron fluctuations. The waves occur at the sharpest density gradient on the low-density side of the boundary [indicated by the yellow shaded region in panels (**a**–**c**)], and the electrons move together with the waves (i.e., are approximately frozen in). The spacecraft moves from the magnetosheath to the magnetosphere, and the neutral point is indicated (*B*_*L*_ = 0, dashed magenta line). The data are displayed in LMN coordinates (see text). **a** The magnitude and components of the magnetic field **B**. **b** Electron density *n*_*e*_. **c** Electric field **E** perpendicular to **B** in the LMN-directions (*E*_*L*⊥_, *E*_*M*⊥_, and *E*_*N*⊥_) and parallel to **B** (*E*_∥_). **d** Electron bulk velocity in the same directions. **e** **E** and the ion and electron convection terms in the **M** direction. **f** Normalized density fluctuations *δn*_*e*_/*n*_*e*_. The cyan dashed line indicates the magnetospheric separatrix, which is the boundary between magnetospheric and reconnected field lines. The separatrix was identified by the strong electron jet directed away from the X line, as described in ref. ^28^ and seen in panel (**d**).

A region where reconnecting current sheets and potential anomalous effects can be found is the terrestrial equatorial magnetopause, the boundary between the shocked solar wind in the magnetosheath and the magnetosphere (Fig. 1a). Magnetic reconnection occurs between the high-density magnetosheath and the more tenuous magnetosphere. This results in reconnection being asymmetric with strong density gradients across the boundary. Figure 1b shows the result from a numerical simulation (see Methods, subsection Simulation description) designed to illustrate the magnetopause reconnection event presented in Fig. 2. The approximate orbit of MMS moving from the magnetosheath to the magnetosphere is indicated, with the turbulent magnetopause separating the two regions. The density fluctuations on the low-density side of the reconnection region are due to lower hybrid waves, which are driven by the strong density gradients in this region.

Figure 2 provides an overview of a magnetopause reconnection event observed by MMS. We use MMS electric^22,23^ and magnetic field data^24,25^, and electron and ion data^26^. In particular, to investigate fluctuations in the electron and ion distributions associated with waves, we use particle moments sampled at 7.5 and 37.5 ms, respectively^27^. The electron sampling rate is high enough to resolve the local lower hybrid frequency and is unique and essential for comparisons with the lower hybrid waves.

Magnetic field data from one MMS spacecraft is shown in Fig. 2a in a local current sheet coordinate system: the current sheet normal points along **N**, **L** is along the anti-parallel magnetic field direction, and **M** = **N** × **L** completes the right-hand coordinate system. The local coordinates are determined using a minimum variance analysis of **B**. The magnetopause crossing is characterized by a reversal in *B*_*L*_ from negative in the high-density magnetosheath to positive in the low-density magnetosphere (Fig. 2b). MMS crosses the magnetopause close to, but southward, of the electron diffusion region (EDR), as indicated by the electron and ion jets reported previously in ref. ^28^. Based on four-spacecraft observations, we estimate the current sheet velocity to be ≈40 km s^−1^ sunward in the **N** direction.

The components of the electric field **E** perpendicular and parallel to the magnetic field **B** are shown in Fig. 2c. The most intense waves are observed on the low-density side of the current sheet, mainly perpendicular to **B** in the **N** and **M** directions, with some intermittent smaller-amplitude higher-frequency fluctuations parallel to **B** (close to the **L** direction). We identify the waves as lower hybrid drift waves driven by the diamagnetic current at the density gradient (Fig. 2b). Lower hybrid waves occur between the ion and electron gyrofrequencies. The waves have a frequency of around ~10 Hz, a phase speed of *v*_ph_ ≈ 140 km s^−1^, and a wavenumber of *kρ*_*e*_ ≈ 0.4^28^, where *ρ*_*e*_ is the thermal electron gyroradius. The analysis techniques used to determine the wave properties are detailed in ref. ^20^. These waves have been proposed as a source of anomalous resistivity and can be important for magnetic reconnection^29^. Some recent studies concluded that the waves are relatively unimportant^5,30,31^, while others conclude that the waves are important for ongoing reconnection^9,12,32^.

Figure 2d shows the perpendicular and parallel components of **V**_*e*_ of the lower hybrid waves. The fluctuations are well resolved and the electron moments can be used to calculate the associated anomalous terms. Large **V**_*e*_ fluctuations are observed not only in the perpendicular but also in the direction parallel to **B**, indicating that the wave vector is not exactly perpendicular to **B**. This means the waves can potentially heat electrons. Figure 2e shows **E** and the electron and ion convection terms −**V**_*e*_ × **B** and −**V**_*i*_ × **B** in the **M** direction. Throughout the interval **E** ≈ −**V**_*e*_ × **B** meaning the electrons move together with the magnetic field (are approximately frozen in) as expected for lower hybrid waves. In contrast, −**V**_*i*_ × **B** remains close to zero. Although ion moments do not fully resolve the waves, this is consistent with the ions being unmagnetized, with only small perturbations in **V**_*i*_. This results in large-amplitude fluctuating currents, which are in turn responsible for fluctuations in **B** (Fig. 2a). Figure 2f displays density fluctuations normalized to the background density, associated with the waves. Large normalized perturbations, *δn*_*e*_/*n*_*e*_ > 0.1, and electric field fluctuations suggest that anomalous resistivity may be significant.

### Anomalous terms associated with waves

To evaluate the effects of waves on the plasma we divide the quantities into fluctuating and quasi-stationary components, *Q* = 〈*Q*〉 + *δQ* where 〈*Q*〉 corresponds to spatial or temporal averaging over fast fluctuations and *δQ* corresponds to fluctuations. Anomalous resistivity is effectively a force on charged particles due to waves, so we analyse a momentum equation. The electron momentum equation for a collisionless plasma is

1 $${m}_{e}\frac{\partial \left({n}_{e}{{{{{{{{\bf{V}}}}}}}}}_{e}\right)}{\partial t}+{m}_{e}\nabla \cdot \left({n}_{e}{{{{{{{{\bf{V}}}}}}}}}_{e}{{{{{{{{\bf{V}}}}}}}}}_{e}\right)+\nabla \cdot {{{{{{{{\bf{P}}}}}}}}}_{e}+{n}_{e}e\left({{{{{{{\bf{E}}}}}}}}+{{{{{{{{\bf{V}}}}}}}}}_{e}\times {{{{{{{\bf{B}}}}}}}}\right)=0,$$

where *e*, *n*_*e*_, *m*_*e*_, **V**_*e*_, and **P**_*e*_ are the unit charge, electron density, mass, bulk velocity, and pressure tensor, respectively, and **E** and **B** are the electric and magnetic fields. Introducing fluctuations, neglecting time derivatives, and averaging yields

2 $$\langle {{{{{{{\bf{E}}}}}}}}\rangle +\langle {{{{{{{{\bf{V}}}}}}}}}_{e}\rangle \times \langle {{{{{{{\bf{B}}}}}}}}\rangle =-\frac{\nabla \cdot \langle {{{{{{{{\bf{P}}}}}}}}}_{e}\rangle }{\langle {n}_{e}\rangle e}-\frac{{m}_{e}}{\langle {n}_{e}\rangle e}\nabla \cdot \left(\langle {n}_{e}\rangle \langle {{{{{{{{\bf{V}}}}}}}}}_{e}\rangle \langle {{{{{{{{\bf{V}}}}}}}}}_{e}\rangle \right)+{{{{{{{\bf{D}}}}}}}}+{{{{{{{\bf{T}}}}}}}}+{{{{{{{\bf{I}}}}}}}}.$$

Here **D**, **T**, and **I** are the anomalous drag (sometimes called resistivity), anomalous viscosity (momentum transport), and anomalous Reynold’s stress, respectively. These quantities are defined as

3 $${{{{{{{\bf{D}}}}}}}}=-\frac{\langle \delta {n}_{e}\delta {{{{{{{\bf{E}}}}}}}}\rangle }{\langle {n}_{e}\rangle },$$

4 $${{{{{{{\bf{T}}}}}}}}=-\frac{\langle {n}_{e}{{{{{{{{\bf{V}}}}}}}}}_{e}\times {{{{{{{\bf{B}}}}}}}}\rangle }{\langle {n}_{e}\rangle }+\langle {{{{{{{{\bf{V}}}}}}}}}_{e}\rangle \times \langle {{{{{{{\bf{B}}}}}}}}\rangle ,$$

5 $${{{{{{{\bf{I}}}}}}}}=-\frac{{m}_{e}}{e\langle {n}_{e}\rangle }\left[\nabla \cdot \left({n}_{e}{{{{{{{{\bf{V}}}}}}}}}_{e}{{{{{{{{\bf{V}}}}}}}}}_{e}\right)-\nabla \cdot \left(\langle {n}_{e}\rangle \langle {{{{{{{{\bf{V}}}}}}}}}_{e}\rangle \langle {{{{{{{{\bf{V}}}}}}}}}_{e}\rangle \right)\right].$$

We define the total anomalous contribution to equation (2) as **R** = **D** + **T** + **I**. We find that the contributions of **I** are negligible compared with **D** and **T**, so they are neglected in the following analyses (see Methods, subsection Estimating the anomalous terms for an example and details).

We study the electron continuity equation to find anomalous flows due to fluctuations

6 $${{{{{{{{\bf{V}}}}}}}}}_{{{{{{{{\rm{anom}}}}}}}}}=\frac{\langle \delta {n}_{e}\delta {{{{{{{{\bf{V}}}}}}}}}_{e}\rangle }{\langle {n}_{e}\rangle }.$$

A cross-field diffusion coefficient *D*_⊥_ relates the electron density and velocity fluctuations to the density gradient in the direction normal to the boundary

7 $${D}_{\perp }=-\frac{\langle \delta {n}_{e}\delta {V}_{e,N}\rangle }{\nabla {\langle {n}_{e}\rangle }_{N}}.$$

A gradient relaxation timescale can be estimated as

8 $${\tau }_{n}={\left(\frac{1}{{n}_{e}}\frac{\partial {n}_{e}}{\partial t}\right)}^{-1}\approx {\left[\frac{\partial }{\partial N}\left(\frac{{D}_{M}}{\langle | {{{{{{{\bf{B}}}}}}}}| \rangle }\right)\right]}^{-1}.$$

For lower hybrid waves, it has not been previously possible from observations to directly evaluate the terms involving electron density or velocity fluctuations, such as 〈*δn*_*e*_*δ***E**〉.

### Anomalous contributions from lower hybrid waves

**Fig. 3:**
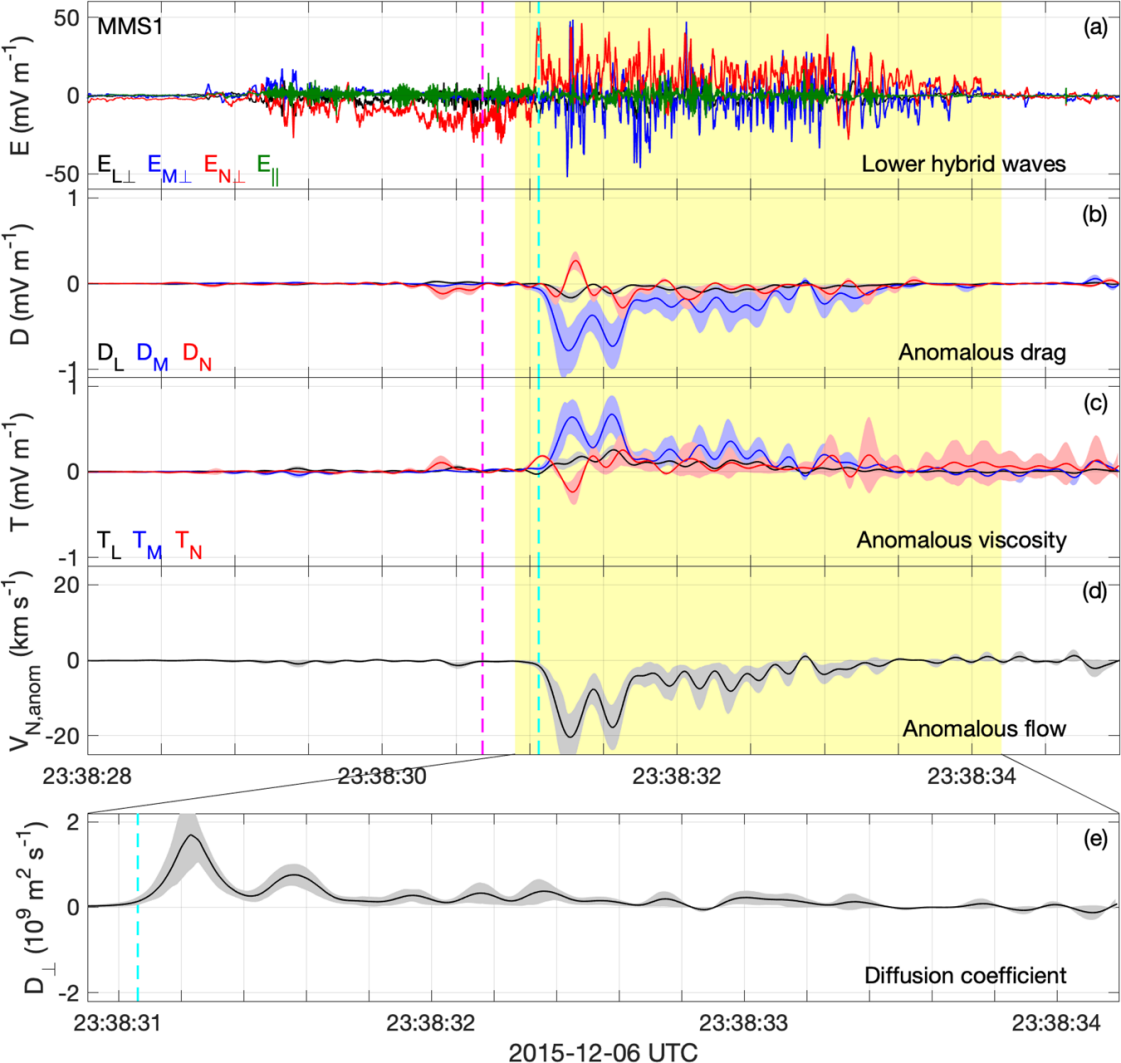
Lower hybrid waves and anomalous terms. Observations of lower hybrid waves and parameters describing anomalous plasma phenomena observed at the magnetopause. The anomalous terms are calculated using all four MMS spacecraft (see Methods, subsection Estimating the anomalous terms). The anomalous terms are negligible at the neutral point (magenta dashed line), and significant when lower hybrid waves are present. The separatrix is indicated by the cyan line. The anomalous drag and viscosity essentially cancel leaving no significant contribution to the reconnection electric field, while there is significant anomalous flow from high to low density, corresponding to a large diffusion coefficient. **a** Perpendicular and parallel components of **E** of lower hybrid waves in LMN coordinates (Fig. 2c) observed by MMS1. **b**, **c** Anomalous drag and viscosity, **D** and **T**, in LMN coordinates. **d** Anomalous flow *V*_N,anom_ in the normal direction. The yellow shaded region in panels (**a**–**d**) indicates the interval when lower hybrid waves are observed. **e** Diffusion coefficient *D*_⊥_. The colored shaded regions associated with the anomalous terms indicate the uncertainty in the calculation based on the uncertainties in the particle moments and electric field (see Methods, subsection Estimating the anomalous terms).

Figure 3a shows the lower hybrid waves from one MMS spacecraft and Fig. 3b–e display the anomalous terms **D**, **T**, anomalous electron flow *V*_N,anom_ in the **N** direction, and the diffusion coefficient *D*_⊥_ in the **N** direction, obtained by combining data from all four spacecraft (see Methods, subsection Estimating the anomalous terms). The terms **D** and **T** have a maximum amplitude of 0.8 ± 0.2 mV m^−1^ (Fig. 3b, c), a small fraction (~2%) of the amplitude of the waves. For comparison, the reconnection electric field associated with magnetopause reconnection is expected to be ~1 mV m^−1^ for fast reconnection, comparable to the peak magnitudes of **D** and **T**. Both **D** and **T** are predominantly in the **M** direction and **D** ≈ −**T**. This is similar to the result found from the simulation in ref. ^33^. The directions of **D** and **T** remain the same while the lower hybrid waves are observed, although some residual fluctuations remain. These fluctuations result from the four-spacecraft averaging used to approximate the spatial averaging needed to compute **D** and **T** and provide an indicator of the uncertainty in the averaging. Similarly, the magnitudes of **D** and **T** are larger than the estimated uncertainties based on the fields and particle measurements (indicated by the shaded regions associated with each anomalous term). The anomalous terms are significant only when large-amplitude waves are present and are localized to the density gradients on the low-density side of the boundary. Thus, the anomalous terms are negligible at the neutral point (*B*_*L*_ = 0). Overall, the contribution to the reconnection electric field is small because **R** ≈ 0, which results from **E** ≈ −**V**_*e*_ × **B** for lower hybrid waves, and the waves do not penetrate into the center of the current sheet.

Figure 3d shows a significant anomalous electron flow *V*_N,anom_ with a magnitude up to 20 km s^−1^ toward the lower-density side. Due to electrons being approximately frozen in **V**_anom_ ≈ −**D** × 〈**B**〉/〈∣**B**∣〉^2^. Figure 3e shows a related large diffusion coefficient *D*_⊥_, which peaks at about 10^9^ m^2^ s^−1^, suggesting that significant broadening of the current layer can occur. Overall, the anomalous electric field [equation (2)] is not likely to affect the reconnection electric field and the reconnection rate. Rather, the lower hybrid waves can produce anomalous diffusion of electrons from higher to lower density regions, thus broadening the current layer. This can in turn affect the reconnection process by modifying the Hall electric and magnetic fields and contributing to the electron heating observed in the magnetospheric inflow region^17,31^. From equation (8) the estimated relaxation time is ~1 s (comparable to the ion-cyclotron period).

### Examples of anomalous terms from lower hybrid waves

**Fig. 4:**
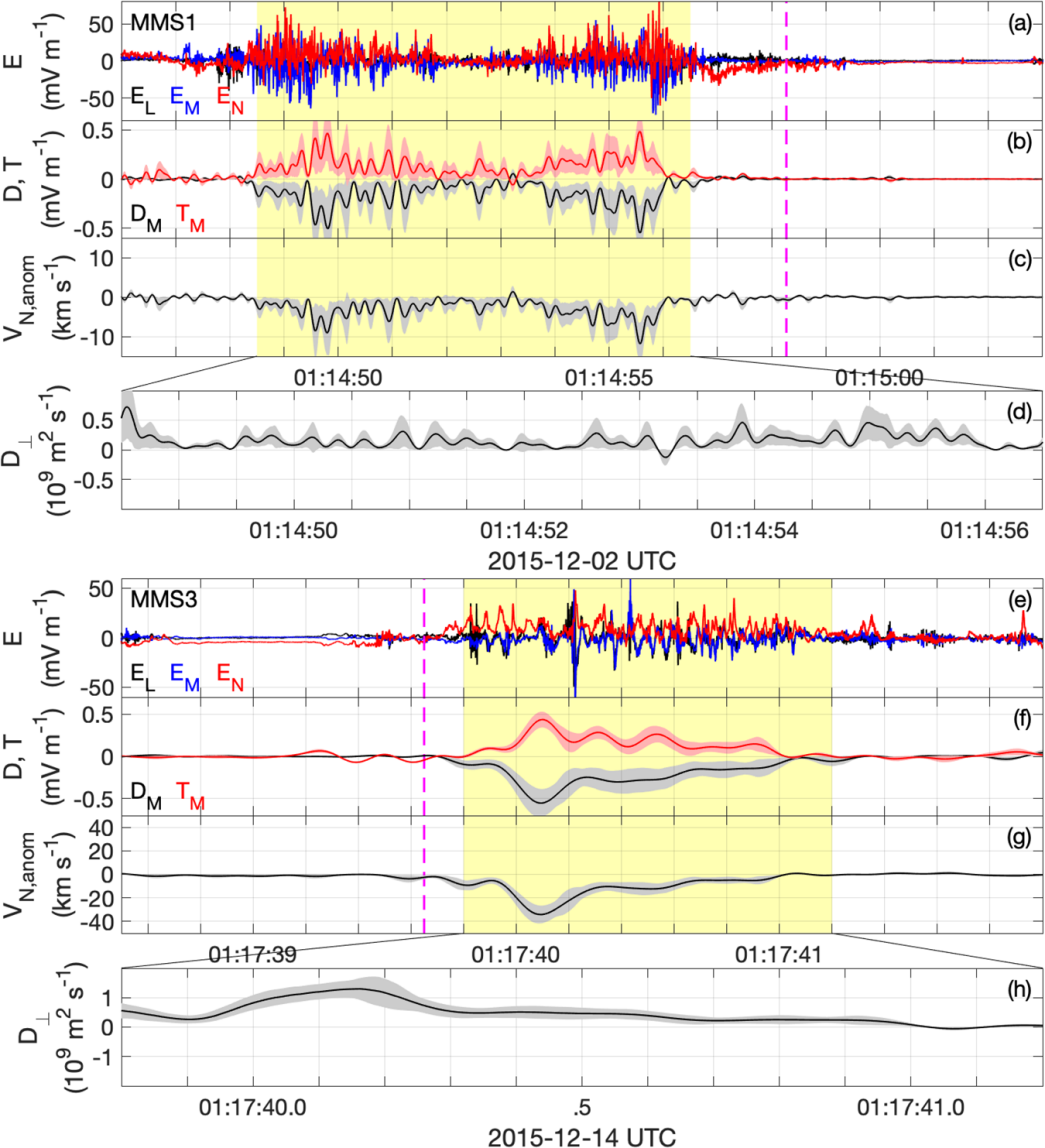
Two magnetopause crossings. Two examples of magnetopause crossings near the reconnection diffusion region and the anomalous terms associated with the lower hybrid waves. In both cases, lower hybrid waves are observed on the low-density magnetospheric side of the current sheet (indicated by the yellow shaded regions). The anomalous terms are computed using the same procedure as in Fig. 3. The first event was observed on 02 December 2015 [panels (**a**–**d**)] and is a magnetopause crossing from the magnetosphere to the magnetosheath. The second event was observed on 14 December 2015 [panels (**e**–**g**)] and is a magnetopause crossing near the electron diffusion region from the magnetosheath to the magnetosphere. In both cases, **R** = **D** + **T** ≈ 0, while large *V*_N,anom_ are observed. **a** Electric field from MMS1 in LMN coordinates. **b** **D**_*M*_ (black) and **T**_*M*_ (red). **c** *V*_N,anom_. **d** *D*_⊥_. Panels (**e**–**h**) plot the same quantities as panels (**a**–**d**), except MMS3 data is plotted in panel (**e**). The colored shaded regions associated with the anomalous terms indicate the uncertainties of the anomalous terms (see Methods, subsection Estimating the anomalous terms).

Figure 4 shows two different magnetopause crossings observed on 02 December 2015 (Fig. 4a–d) and 14 December 2015 (Fig. 4e–h). In Fig. 4a–d the spacecraft crossed from the magnetosphere to the magnetosheath. The spacecraft crossed the EDR at around 01:14:56 UT, close to the neutral point^34^. The lower hybrid waves are observed for ≈10 s on the magnetospheric side of the boundary. In Fig. 4e–h the spacecraft crossed the EDR from the magnetosheath and magnetosphere^35,36^, with the lower hybrid waves observed on the magnetospheric side for 1 s. The properties of the lower hybrid waves were investigated in detail in ref. ^20^.

Overall, the properties of the magnetopause crossings are similar to the event in Figs. 2, 3. Namely, large-amplitude lower hybrid waves are observed on the magnetospheric side, *D*_*M*_ < 0 and *T*_*M*_ > 0, such that **D** + **T** is small, and a significant *V*_N,anom_ < 0 is observed. For the 14 December 2015 event **D** and **T** have a significant component in the **L** direction due to the significant *B*_*M*_ (guide field). In both events, the anomalous terms are negligible at the neutral point (indicated by the magenta lines in Fig. 4).

Although the amplitude of the lower hybrid waves are comparable in these two events, ∣*V*_N,anom_∣ is significantly larger for the 14 December 2015 event. This is likely because **B** is smaller, corresponding to a larger amplitude *δ***V**_*e*,⊥_ ≈ *δ***E** × **B**/∣**B**∣^2^. At the times when ∣*V*_N,anom_∣ peaks we estimate *D*_⊥_ = 0.58 × 10^9^ m^2^ s^−1^ and *D*_⊥_ = 1.21 × 10^9^ m^2^ s^−1^, respectively, for the 02 December 2015 and 14 December 2015 events. This corresponds to the diffusion of electrons across **B** from the magnetosheath to the magnetosphere in both cases. Thus the diffusion coefficients are significant and comparable to the values obtained in Fig. 2. In both cases, the uncertainties are smaller than the peak values of the anomalous terms. To summarize, the two events presented in Fig. 4 show the same qualitative behavior as the 06 December 2015 event: **D** and **T** both reach about 0.5 mV m^−1^ but have opposite signs so the contribution to **E** is small. Large anomalous flows and cross-field electron diffusion toward the magnetosphere are observed. In all three cases the peak values of ∣**D**∣ and ∣**T**∣ are ~2% of the maximum amplitude of *δ***E**.

### Statistical results

**Fig. 5:**
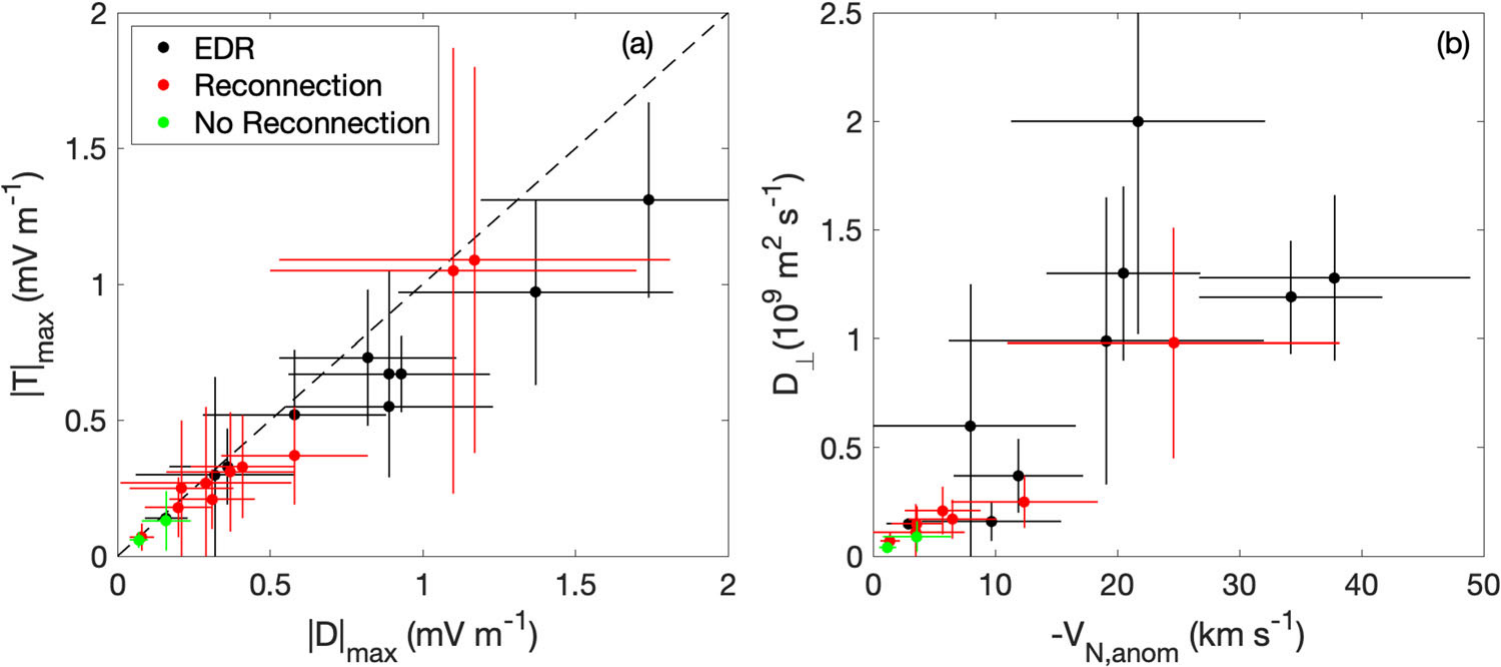
Statistics of anomalous terms. Anomalous terms are calculated from 22 magnetopause current sheets with lower hybrid waves. The black points indicate EDR crossings, red points indicate reconnection crossings outside the EDR, and green points indicate boundary crossings without clear evidence of reconnection. The maximum values of the anomalous viscosity and anomalous drag for each event, ($| {{{{{{{\bf{T}}}}}}}}{| }_{\max }$ and $| {{{{{{{\bf{D}}}}}}}}{| }_{\max }$), are comparable. The diffusion coefficient *D*_⊥_ tends to increase with the negative of the anomalous flow *V*_N,anom_ up to tens of km s^−1^, indicating significant flow from high to low density in the direction normal for large *D*_⊥_. **a** $| {{{{{{{\bf{T}}}}}}}}{| }_{\max }$ versus $| {{{{{{{\bf{D}}}}}}}}{| }_{\max }$, **b** *D*_⊥_ versus − *V*_N,anom_. The dashed line in panel (**a**) indicates $| {{{{{{{\bf{D}}}}}}}}{| }_{\max }=| {{{{{{{\bf{T}}}}}}}}{| }_{\max }$. The horizontal and vertical lines indicate the uncertainties of $| {{{{{{{\bf{D}}}}}}}}{| }_{\max }$ and $| {{{{{{{\bf{T}}}}}}}}{| }_{\max }$ in (**a**) and *V*_N,anom_ and *D*_⊥_ in (**b**). See Methods, subsection Estimating the anomalous terms for details on the calculation of the uncertainties.

Figure 5 shows statistics from magnetopause crossings where high-resolution particle moments are available and lower hybrid waves are observed. We divide each event into (1) EDR crossings, where the waves are observed adjacent to EDR regions identified in ref. ^37^. (2) Reconnection events, where the waves are observed at boundaries where reconnection signatures, such as ion outflows, are observed. (3) Non-reconnection events, where no clear reconnection signatures are observed.

In each case the largest **D** was in the −**M** direction and the largest **T** was in the **M** direction. Figure 5a shows the maximum **T** ($| {{{{{{{\bf{T}}}}}}}}{| }_{\max }$) versus the maximum **D** ($| {{{{{{{\bf{D}}}}}}}}{| }_{\max }$) and the associated uncertainties for each event. Here $| {{{{{{{\bf{D}}}}}}}}{| }_{\max }$ and $| {{{{{{{\bf{T}}}}}}}}{| }_{\max }$ can reach ≈1.5 mV m^−1^, with $| {{{{{{{\bf{T}}}}}}}}{| }_{\max }$ increasing approximately linearly with $| {{{{{{{\bf{D}}}}}}}}{| }_{\max }$. Both peak at approximately the same time, so for all cases **R** ≈ 0. We find that $| {{{{{{{\bf{T}}}}}}}}{| }_{\max }$ tends to be slightly smaller than $| {{{{{{{\bf{D}}}}}}}}{| }_{\max }$, possibly due to small deviations from the frozen-in condition for electrons due to fluctuations in the electron pressure due to density fluctuations. The largest **D** and **T** correspond to magnetic reconnection events and EDRs, although more non-reconnection events need to be analyzed.

Figure 5b shows the values of *D*_⊥_ where ∣*V*_N,anom_∣ peaks versus the maximum −*V*_N,anom_. Here *D*_⊥_ tends to increase as −*V*_N,anom_ increases. Each case corresponds to diffusion from higher to lower densities. We find that *D*_⊥_ ranges from 0.05 × 10^9^ to 2 × 10^9^ m^2^ s^−1^, i.e., from small to very significant diffusion^38,39^. The largest −*V*_N,anom_ and *D*_⊥_ tend to occur close to EDRs, although there are cases where −*V*_N,anom_ and *D*_⊥_ are also small near EDRs. Thus, cross-field diffusion and associated broadening are expected to be highly variable during magnetopause reconnection. The results suggest that *D*_⊥_ may be the largest close to the EDR, although further work and more events are required to confirm this.

## Discussion

We find that for lower hybrid waves the anomalous terms **D**, **T**, and **V**_anom_ can be accurately determined from the data and that **R** = **D** + **T** ≈ 0, so the contribution to the reconnecting electric field is negligible because electrons are approximately frozen in. However, the diffusion coefficient *D*_⊥_ and *V*_N,anom_ can often be significant, corresponding to transport from the higher-density magnetosheath to the lower-density magnetosphere, producing significant broadening of the magnetopause density gradient.

Overall, these direct observations of anomalous terms in collisionless plasma open a new window to investigate fundamental plasma physics. Directly evaluating all terms involved in wave-particle interactions will show which processes are important, and which are not, in many astrophysical plasmas. In many reconnection events, the lower hybrid waves are observed over several seconds at large amplitude, which suggests that the density gradient is driven by ongoing reconnection, while lower hybrid waves counter this.

## Methods

### Estimating the anomalous terms

For each event we rotate the vector quantities into LMN coordinates, where **N** is normal to the magnetopause pointing sunward, **L** is along the reconnecting magnetic field direction, and **M** completes the coordinate system and is close to the guide-field direction. We determine the coordinate system using a minimum variance analysis of **B** across the magnetopause. The reliability of the **N** direction is confirmed by determining the boundary normal velocity using four-spacecraft timing analysis, as well as minimum variance analysis of the current density **J**. In most cases, the uncertainty in the coordinate system directions are small and do not significantly affect the results.

Ideally, the quantities in equation (2) are computed from an ensemble average in the **M** direction. With MMS we must use a four-spacecraft average to estimate these quantities. For all events, the spacecraft were in tetrahedral configurations with spacecraft separations ranging from ~15 to ~5 km. These separations are well below ion spatial scales at the magnetopause, but larger than electron spatial scales, which is ideal for studying lower hybrid waves. To calculate the anomalous and background quantities we use the following procedure:

1. We resample all field data to the sampling frequency of the high-resolution (7.5 ms) electron moments and perform a four-spacecraft timing analysis on *B*_*L*_ at the current sheet to determine the boundary normal velocity and the time delays between the spacecraft. Typical boundary normal speeds range from ~10 to ~100 km s^−1^.
2. We use the time delays to offset the spacecraft times so all spacecraft cross the boundary layer at the same time as MMS1.
3. To obtain the non-fluctuating terms 〈*Q*〉 we average the time-shifted quantities over the four spacecraft and bandpass filter below 5 Hz. At the magnetopause the lower hybrid waves are typically found at frequencies 10 Hz < *f* < 30 Hz.
4. To obtain *δQ* associated with the lower hybrid wave fluctuations we bandpass filter *Q* above 5 Hz. The specific bandpass frequency does not significantly modify the results, as long as it is not too high to significantly remove lower hybrid wave power.
5. We obtain 〈*δQ*_1_*δQ*_2_〉 by averaging *δQ*_1_*δQ*_2_ over the four spacecraft then low-pass filter the result below 5 Hz to remove any remaining higher-frequency fluctuating components.

To evaluate equation (4) we expand it to obtain

9 $${T}_{L}=\langle \delta {V}_{\!\!N}\delta {B}_{M}\rangle -\langle \delta {V}_{\!\!M}\delta {B}_{N}\rangle +\left[\frac{\langle \delta {n}_{\!e}\delta {V}_{\!\!N}\rangle \langle {B}_{\!M}\rangle +\langle \delta {n}_{\!e}\delta {B}_{\!M}\rangle \langle {V}_{\!\!N}\rangle -\langle \delta {n}_{e}\delta {V}_{\!\!M}\rangle \langle {B}_{N}\rangle -\langle \delta {n}_{\!e}\delta {B}_{N}\rangle \langle {V}_{\!\!M}\rangle }{\langle {n}_{\!e}\rangle }\right],$$

10 $${T}_{\!\!M}=\langle \delta {V}_{\!\!L}\delta {B}_{N}\rangle -\langle \delta {V}_{\!\!N}\delta {B}_{L}\rangle +\left[\frac{\langle \delta {n}_{\!e}\delta {V}_{\!\!L}\rangle \langle {B}_{N}\rangle +\langle \delta {n}_{\!e}\delta {B}_{N}\rangle \langle {V}_{\!\!L}\rangle -\langle \delta {n}_{\!e}\delta {V}_{\!\!N}\rangle \langle {B}_{L}\rangle -\langle \delta {n}_{\!e}\delta {B}_{L}\rangle \langle {V}_{\!\!N}\rangle }{\langle {n}_{\!e}\rangle }\right],$$

11 $${T}_{\!\!N}=\langle \delta {V}_{\!\!M}\delta {B}_{L}\rangle -\langle \delta {V}_{\!\!L}\delta {B}_{\!M}\rangle +\left[\frac{\langle \delta {n}_{\!e}\delta {V}_{\!\!M}\rangle \langle {B}_{L}\rangle +\langle \delta {n}_{\!e}\delta {B}_{L}\rangle \langle {V}_{\!\!M}\rangle -\langle \delta {n}_{\!e}\delta {V}_{\!\!L}\rangle \langle {B}_{M}\rangle -\langle \delta {n}_{\!e}\delta {B}_{M}\rangle \langle {V}_{\!\!L}\rangle }{\langle {n}_{\!e}\rangle }\right].$$

All terms are calculated to determine **T**, although we find that only the components involving 〈*δn*_*e*_*δ***V**_*e*_〉 are significant. The uncertainties in the anomalous terms are calculated from the uncertainties in the electron moments and assuming a 10% uncertainty in the gain of the electric field. The uncertainty in the electric field is based is on the fact that the gain is validated by comparing the electric field with the DC convection field caused by the spacecraft moving relative to a magnetized plasma, and there can be small changes in the gain due to changes in the plasma conditions. The uncertainties in the electron moments are based on the counting statistics of the particle distributions. This is only available at 30 ms sampling, so we assume that the uncertainties are four times larger for the 7.5 ms moments we use, due to the reduced azimuthal sampling. The magnitude of the uncertainties of the particle moments are compared with the magnitude of the envelope of the fluctuating quantities to estimate the relative uncertainties.

Estimates of the anomalous contributions from the electron inertial term and time derivative in equation (1) indicate that they are much smaller than **D** and **T** due to the *m*_*e*_/*e* dependence. The **M** component of the anomalous inertial terms (anomalous Reynold’s stress^5^) **I** can be well approximated by assuming that the anomalous terms in **I** vary primarily in the **N** direction, which is given by

12 $${I}_{M}=-\frac{{m}_{e}}{e\langle {n}_{e}\rangle }\frac{\partial }{\partial N}\left(\langle {n}_{e}\rangle \langle \delta {V}_{M}\delta {V}_{N}\rangle +\langle {V}_{M}\rangle \langle \delta {n}_{e}\delta {V}_{N}\rangle \right).$$

Since the method of obtaining the anomalous terms equation (12) relies on four-spacecraft averaging, the four spacecraft cannot be used to calculate the gradient associated with these terms. Therefore, the gradient is approximated assuming these quantities move past the spacecraft at the boundary normal velocity, such that ∂*N* = −*v*_*N*_∂*t*, where *v*_*N*_ is the boundary normal velocity estimated from the four-spacecraft timing of the current sheet. The values of *I*_*M*_ obtained from equation (12) are significantly smaller than **D** and **T** and do not significantly contribute to **R**.

**Fig. 6:**
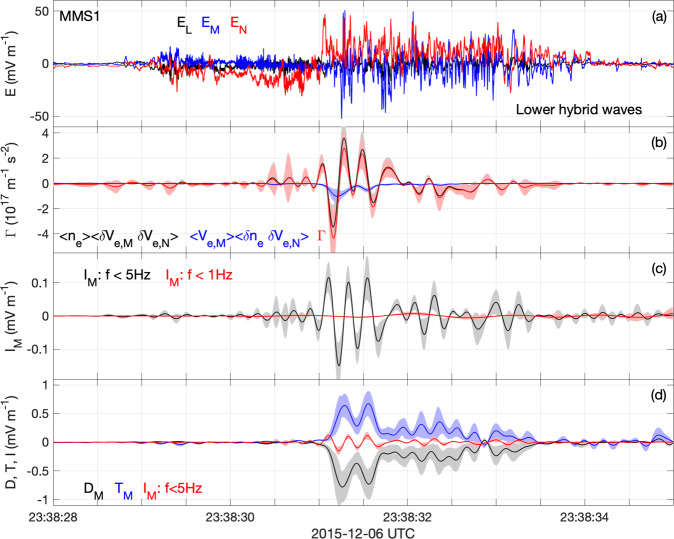
Anomalous inertial electric field. Estimate of the anomalous inertial electric field for the lower hybrid waves observed at 06 December 2015 magnetopause crossing. **a** Electric field of lower hybrid waves from MMS1 in LMN coordinates. **b** The anomalous terms 〈*n*_*e*_〉〈*δV*_*M*_*δV*_*N*_〉 (black), 〈*V*_*M*_〉〈*δn*_*e*_*δV*_*N*_〉 (blue), and Γ = 〈*n*_*e*_〉〈*δV*_*M*_*δV*_*N*_〉 + 〈*V*_*M*_〉〈*δn*_*e*_*δV*_*N*_〉 (red). **c** *I*_*M*_ where the nominal 5 Hz low-pass filter is used (black) and where a 1 Hz low-pass filter is used (red). **d** *D*_*M*_ (black), *T*_*M*_ (blue), and *I*_*M*_ for the 5 Hz low-pass filter (red). The colored shaded regions associated with the anomalous terms indicate the uncertainty in the calculation based on the particle moments.

As an example, Fig. 6 shows *I*_*M*_ estimated from equation (12) and a comparison with *D*_*M*_ and *T*_*M*_ for the 06 December 2015 event (Figs. 2, 3). Figure 6a shows the electric field associated with the lower hybrid waves from MMS1. In Fig. 6b we plot the anomalous terms 〈*n*_*e*_〉〈*δV*_*M*_*δV*_*N*_〉, 〈*V*_*M*_〉〈*δn*_*e*_*δV*_*N*_〉, and Γ = 〈*n*_*e*_〉〈*δV*_*M*_*δV*_*N*_〉 + 〈*V*_*M*_〉〈*δn*_*e*_*δV*_*N*_〉. We find that 〈*V*_*M*_〉〈*δn*_*e*_*δV*_*N*_〉 < 0 due the term being proportional to *V*_N,anom_. In contrast, 〈*n*_*e*_〉〈*δV*_*M*_*δV*_*N*_〉 fluctuates with very little offset from zero. This is due to the lack of consistent correlation between the *δV*_*M*_ and *δV*_*N*_ associated with the lower hybrid waves. As a result, Γ fluctuates and is similar to 〈*n*_*e*_〉〈*δV*_*M*_*δV*_*N*_〉. In Figure 6c we plot *I*_*M*_ for Γ and Γ bandpass filtered below 1 Hz to remove the fluctuations. We find that *I*_*M*_ fluctuates around zero when the 5 Hz low-pass filter is used. There is negligible large-scale offset, as seen for the <1 Hz case. Thus, *I*_*M*_ for the 5 Hz low-pass filter is overestimated. In Fig. 6d we plot *D*_*M*_, *T*_*M*_, and *I*_*M*_ for the 5 Hz bandpass filter. We find that the *I*_*M*_ is much smaller than *D*_*M*_ and *T*_*M*_. Both *D*_*M*_ and *T*_*M*_ have clear background components, in contrast to *I*_*M*_. Similar results are found for the other events, and there is no clear evidence that **I** can significantly contribute to **R** for lower hybrid waves. We conclude that the contribution of *I*_*M*_ to the total anomalous electric field is negligible based on MMS observations.

This differs from the results of three-dimensional simulations^5,9,31,32^, which have found that *I*_*M*_ could be significant. Possible reasons for these differences are:

1. Artificial plasma conditions, such as reduced electron to ion mass ratio and reduced ratio of electron plasma to cyclotron frequency, are needed to run 3D simulations.
2. When spatially averaging over the **M** direction in simulations very low-frequency fluctuations, such as current sheet kinking, are typically included, which can lead to large anomalous terms that are not due to lower hybrid waves^5^. In observations, we used a high-pass filter of 5 Hz, which removes such low-frequency fluctuations, if they are present.

### Simulation description

We model the 06 December 2015 event using the fully kinetic iPIC code^40^. The code uses an implicit moment method, which allows the cell size to exceed the Debye length^41^. The code **x**, **y**, and **z** coordinates point in the **L**, **N**, and −**M** directions used in this letter. The simulation is initialized with two thin current sheets of width 1*d*_*i*_ and 2*d*_*i*_ at *y* = *L*_*y*_/4 and *y* = 3*L*_*y*_/4, respectively, where *d*_*i*_ is the ion inertial length in the magnetosheath. The ion-to-electron mass ratio is *m*_*i*_/*m*_*e*_ = 256 and the speed of light to the reference Alfvén speed ratio is *c*/*V*_*A*_ = 103. The parameters used to set up the asymmetric force balance are [*B*_*L*_, *B*_*M*_, *n*_*e*_, *T*_*e*_, *T*_*i*_] = [−37 nT, −16 nT, 14 cm^−3^, 32 eV, 1200 eV] on the magnetosheath side and [73 nT, −16 nT, 1.85 cm^−3^, 164 eV, 3900 eV] on the magnetospheric side.

The simulation is performed in two steps:

1. Asymmetric magnetic reconnection is first run in two dimensions in **x** − **y** coordinates in a double periodic domain^42^. The size of the domain is *L*_*x*_ × *L*_*y*_ = 2822 × 1058 km^2^ and is resolved by 1728 × 648 cells. A weak localized perturbation at (*L*_*x*_/2, *L*_*y*_/4) is used to initiate reconnection^43^.
2. The three-dimensional (3D) simulation is initialized at time *t*Ω_*ci*_ = 35 once steady-state reconnection is reached, where Ω_*ci*_ is the angular ion-cyclotron frequency. The initial conditions of the 3D simulation are the fields and particle information from the 2D run and replicated in the **z**-direction. The computational domain is *L*_*x*_ × *L*_*y*_ × *Lz* = 2822 × 1058 × 117.5 km^3^ and is resolved by 1728 × 648 × 72 cells. This replicated geometry is suitable for investigating instabilities, such as the lower hybrid drift instability, with wavelengths short compared with *L*_*z*_.

## Data Availability

MMS data were available at https://lasp.colorado.edu/mms/sdc/public. The data can be found in the following directories: mms#/edp/brst/l2/dce/ for electric fields, mms#/fgm/brst/l2/ for the background magnetic field, mms#/scm/brst/l2/scb/ for the fluctuating magnetic field, mms#/fpi/brst/l2/des-moms/ for the background electron moments, mms#/fpi/brst/l2/des-qmoms/ for the highest resolution electron moments, and mms#/fpi/brst/l2/dis-qmoms/ for the highest resolution ion moments. Source data required to generate the figures in this paper can be found at https://github.com/danbgraham/anomres^44^ and are available on request. The datasets generated during and/or analysed during the current study are available from the corresponding author on reasonable request.

## Code availability

All figures and data analyses were performed using the IRFU-Matlab data analysis package, which is available at https://github.com/irfu/irfu-matlab. The scripts and functions required to compute the anomalous terms and reproduce the figures in this paper can be found at https://github.com/danbgraham/anomres^44^. The simulation code is available at https://github.com/CmPA/iPic3D.
